# Rare homozygous mutation in *TUBB8* associated with oocyte maturation defect-2 in a consanguineous mating family

**DOI:** 10.1186/s13048-020-00637-4

**Published:** 2020-04-21

**Authors:** Qiong Xing, Ruyi Wang, Beili Chen, Lin Li, Hong Pan, Tengyan Li, Xu Ma, Yunxia Cao, Binbin Wang

**Affiliations:** 1 Reproductive Medicine Center, Department of Obstetrics and Gynecology, the First Affiliated Hospital of Anhui Medical University, 218 Jixi Road, Shushan, Hefei, 230022 China; 2 Anhui Province Key Laboratory of Reproductive Health and Genetics, Biopreservation and Artificial Organs, Hefei, China; 3 Anhui Provincial Engineering Research Center, Anhui Medical University, Hefei, P.R. China; 4 Graduate School of Peking Union Medical College, Beijing, China; 5 Center for Genetics, National Research Institute for Family Planning, 12 Dahuisi Road, Haidian, Beijing, 100081 China; 6 Central Laboratory, Beijing Obstetrics and Gynecology Hospital, Capital Medical University, Yaojiayuan Road 251, Chaoyang, Beijing, 100026 China

**Keywords:** Oocyte maturation defect-2, *TUBB8*, Whole exome sequencing, Missense mutation, Parthenogenesis

## Abstract

**Purpose:**

Variations in many genes may lead to the occurrence of oocyte maturation defects. To investigate the genetic basis of oocyte maturation defects, we performed clinical and genetic analysis of a pedigree.

**Methods:**

The proband with oocyte maturation defect-2 receiving ovulation induction therapy and her parents were selected for clinical detection, whole exome sequencing and Sanger sequencing. One unrelated healthy woman received ovulation induction therapy as control. Mutations were assessed after frequency screening of public exome databases. Then homozygous variants shared by the proband and her parents were selected.

**Results:**

Arrest of oocytes maturation was observed. A new missense mutation in *TUBB8* (*TUBB8*: NM_177,987: exon 2: c. C161T: p. A54V) was identified, which was shown to be rare compared with public databases. The variant was highly conserved among primates, and was suggested to be deleterious by online software prediction.

**Conclusions:**

The homozygote of this variant (*TUBB8*: NM_ 177,987: exon 2:c.C161T: p.A54V) might affect spindle assembly, cause arrest of oocyte maturation and lead to oocyte maturation defect-2.

## Introduction

In the fetal ovary, oocytes pause at prophase I. When puberty starts, the maturation process of oocytes begins, and oocytes resume meiosis with a surge of luteinizing hormone. Germinal vesicle (GV) breakdown, spindle assembly, chromosomal migration, asymmetric division, and extrusion of the first polar body occur in turn. The oocytes arrest at metaphase II (MII) until fertilization [1, 2]. Primary infertility or failure of in vitro fertilization (IVF) were the clinical manifestation of oocyte maturation defects (OOMDs). Failure of GV breakdown, absence of the first polar body, and failure to progress beyond MII could result in arrest of oocyte maturation [3].

Previous researchers have found many genes related to OOMDs, including *ZP1*, *ZP2*, *ZP3*, *WEE2*, *PATL2*, and *TUBB8*. Mutations in *ZP1*, *ZP2*, and *ZP3* could result in oocyte maturation defect-1 (OOMD1), OOMD6, and OOMD3, respectively. The absence of the zona pellucida was caused by mutations in these three genes and this resulted in degeneration of oocytes and “empty follicle syndrome” during IVF treatment [4–6]. The mutations in *WEE2* resulted in OOMD5, which was characterized by arrest of oocyte maturation at MII and inability to form pronuclei after fertilization [7]. While the mutations in *PATL2* resulted in OOMD4, which was characterized by fertilization failure and early embryonic arrest [8].

Feng et al. discovered that OOMD2 was caused by mutations in the *TUBB8* gene which located at chromosome 10. OOMD2 was characterized by spindle damage in metaphase I (MI) or MII, resulting in fertilization failure [9, 10].

To study the genetic basis of oocyte maturation defects, we found a consanguineous mating pedigree and searched for the pathogenic gene of this family by whole exome sequencing (WES). We targeted the *TUBB8* gene.

## Material and methods

### Participants

**Fig. 1:**
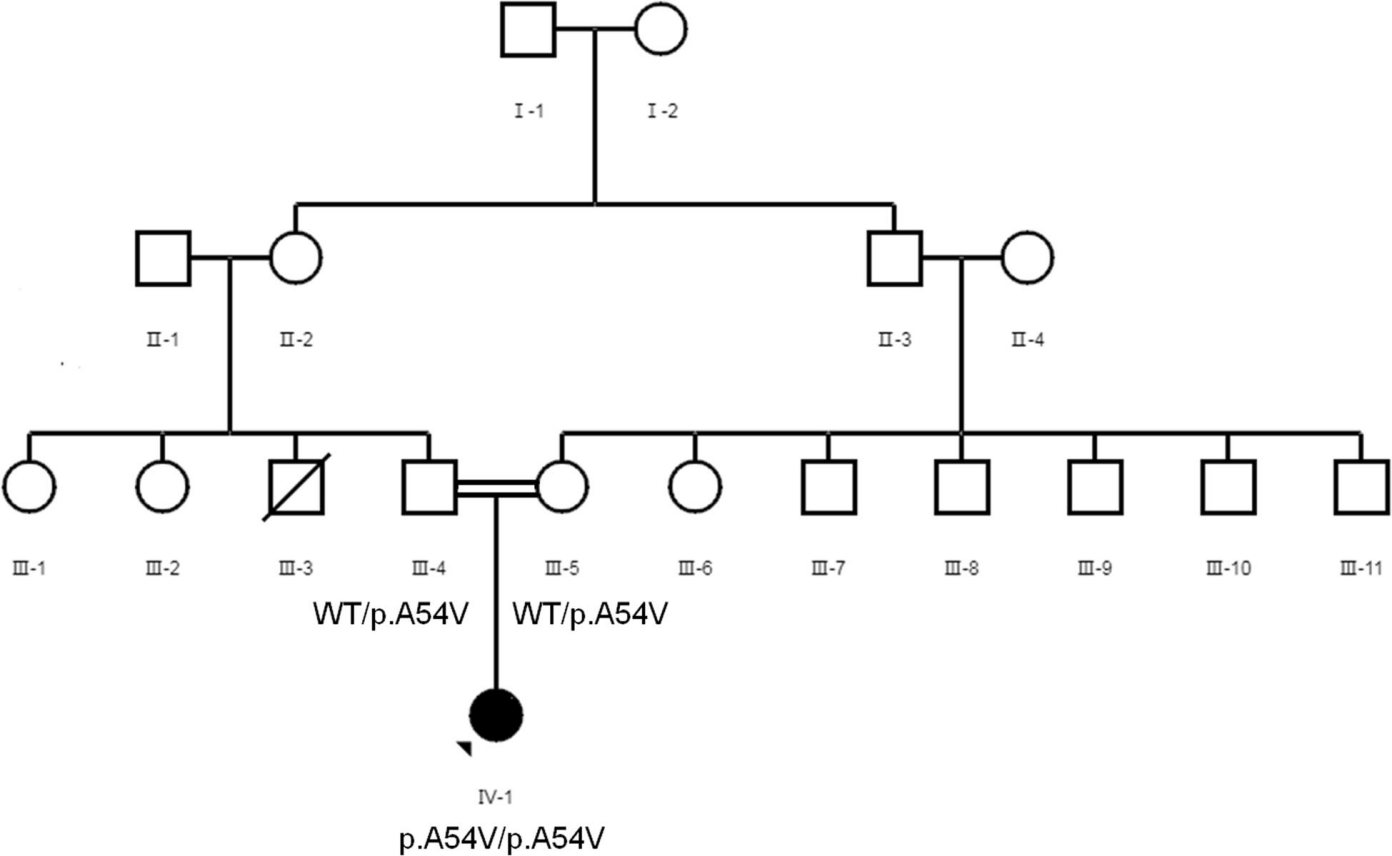
Pedigree chart of the proband’s family with OOMD2

**Fig. 2:**
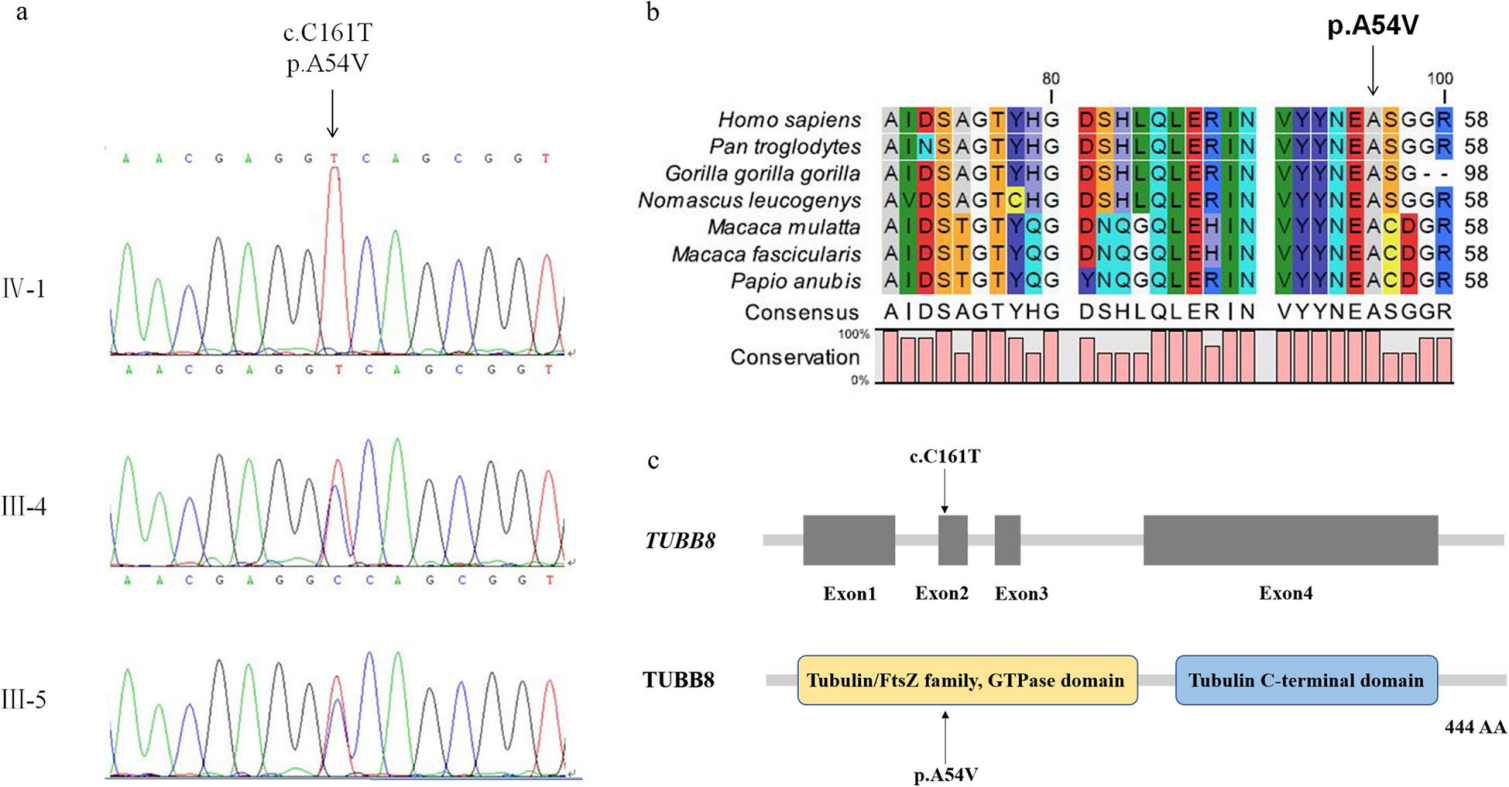
Genetic analysis of a mutation in *TUBB8*. **a** p.A54V is a homozygous mutation with a recessive inheritance pattern. **b** Sanger Sequencing of the proband and her parents. Her parents are carriers of the variant and the proband is a homozygote of the variant. **c** Multiple alignment of *TUBB8* indicates that p.A54V is highly conserved

The family of the proband and an unrelated healthy woman were recruited from the Reproductive Center of the First Affiliated Hospital of Anhui Medical University. The clinical features of the unrelated woman were normal, and most of her oocytes retrieved from body were mature. The proband and her parents (Fig. 1) underwent genetic testing and analysis (Fig. 2). We collected peripheral blood samples from the three individuals. The study protocol was approved by the Ethics Committee of the National Research Institute for Family Planning, and written informed consent was obtained from all participants.

The proband of a consanguineous mating family was affected by OOMD2 with unique clinical characteristics. The proband (IV-1) was a woman with primary infertility whose oocytes showed developmental disorders during induction of clinical ovulation. Her parents were not affected and they were cousins (Fig. 1).

The proband, who was 31 years old, has not got pregnant for 5 years after marriage. She was 163 cm height and weighed 50 kg. Clinical features, routine test results, endocrine test results, immunological test results, and a hysteroscopy examination were all normal. The chromosome examination showed a normal karyotype 46, XX, while her husband showed 46, XY. The proband’s characteristics are shown in Table 1.

**Table 1:** Basal characteristics

Age (years)	Infertility duration (years)	BMI (kg/m^2^)	E2(pmol/L)	FSH (IU/L)	LH (IU/L)	AMH (ng/ml)	PRL (ng/ml)	T (nmol/l)	P (nmol/l)	TSH (mIU/L)
31	5	18.8	153	6.27	3.69	3.6	20.13	2.2	1.69	2.47

*BMI* Body mass index (normal range: 18.5–23.9 kg/m^2^), *E2* Estradiol (normal range: 40–253 pmol/L), *FSH* Follicle stimulating hormone (normal range: 2.5–10.2 IU/L), *LH* Luteinizing hormone (normal range: 1.9–12.5 IU/L), *AMH* Anti-Müllerian hormone (normal range: 0.24–11.78 IU/L), *PRL* Prolactin (normal range: 5.18–26.53 ng/ml), *P* Progesterone (normal range: 0.6–1.9 nmol/l), *T* Testosterone (normal range: 0.9–2.9 nmol/l), *TSH* Thyrotropin (normal range: 0.49–4.67mIU/L)

### Evaluation of oocyte phenotypes

The proband received 3 cycles of controlled ovarian stimulation (COS) during her IVF treatment (Table 1). The first cycle was treated with a long regimen, the second cycle was treated with mild-stimulation protocol and the third cycle was treated with mild-stimulation and in vitro maturation protocols (shown in Table 2). During the whole process of 3 cycles of COS, development of follicles’ size and hormone level were all normal.

**Table 2:** Clinical characteristics of 3 cycles

Cycle number	Date/Menstrual cycle	Medicine	Follicles of right ovary (mm(N))	Follicles of left ovary (mm(N))	Total Number of oocyte retrieved	Stage of Oocyte (N) after 24–36 h of culture in vitro
1					23	MI(22) + GV(1)
	2017.10.25/D18	GnRHa (mg):0.9				
	2017.11.8/D5	rFSH(U): 150*5d	2*2(3)	2*2(4)		
	2017.11.13/D10	rFSH(U): 150*4d	8*8(3),6*6(3)	10*9(2),8*7(1),7*5(3)		
	2017.11.17/D14	rFSH(U): 150*3d	15*14(1),14*14(1),12*11(4)	15*15(1),15*13(2),16*12(1), 14*14(2),14*10(1),12*12(2)		
	2017.11.20/D17	rHCG (ug): 250	21*21(1)	22*19(1),21*20(2),19*17(2), 19*16(2),18*17(1)		
2					21	MI(12) + GV(2)+ + degenerate oocytes (7)
	2018.3.19/D3	HMG(U): 225*5d Clomiphene citrate (mg): 100*5d	5–6(3–6)	6–7(3–6)		
	2018.3.24/D8	HMG(U): 225 Clomiphene citrate (mg): 100	16*13(1),14*13(1),13*12(1)	15*11(2)		
	2018.3.25/D9	HMG(U): 225*2d Clomiphene citrate (mg): 100*2d Ganirelix (mg): 0.25*2d				
	2018.3.27/D11	HMG(U): 225 Ganirelix (mg): 0.25	23*21(1),20*17(1),18*15(2),17*17(2),17*15(2)	20*17(2),18*14(2),17*12(2)		
	2018.3.28/D12	Ganirelix (mg): 0.25 HCG (IU):8000	22*22(1),21*20(1),20*20(3),19*19(2),18*18(2)	23*17(1),22*20(1),18*18(1), 17*17(1)		
3					12	MI(7) + GV(3) + degenerate oocytes(2)
	2018.9.18/D3	HMG(U): 225*4d	3–4(6)	3–4(7)		
	2018.9.22/D7	HMG(U): 225*2d	8*8(1),6*5(1),5*5(1),4*4(1),4*3(1)	10*7(1),9*9(1),9*7(1),8*8(1)		
	2018.9.24/D9	HCG(U): 10000	13*12(1),8*8(2),7*7(1),5*5(4)	13*13(1),13*12(1),12*12(1), 11*11(1),10*10(2),9*9(1)		

*D* Day number of menstrual cycle, *d* Days of continuous medication, *N* Number, *rFSH* recombinant follicle stimulating hormone (Gonal-F, merck Serono, Switzerland), *rHCG* recombinant human chorionic gonadotrophin (Ovidrel, merck Serono, Germany), *HMG* human menopausal gonadotropin (Lebaode, Livzon, Zhuhai), *HCG* human chorionic gonadotrophin (Livzon, Zhuhai), GnRHa (Diphereline, Ipsen, France), Ganirelix (Orgalutran, merck sharp organon, USA), Clomiphene citrate (Clomifene Citrate Tablets, Hengshan, shanghai)

**Fig. 3:**
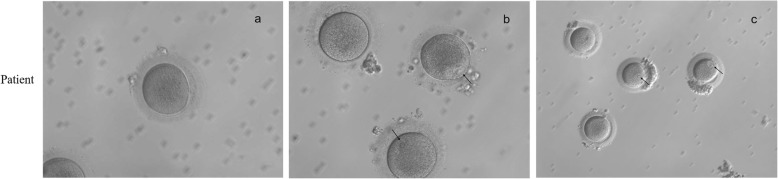
Morphology of oocytes that were retrieved from the patient with a *TUBB8* mutation (p.A54V). **a** One MI oocyte from the patient. **b** One GV oocyte and two MI oocytes from the patient. **c** Two GV oocytes and two MI oocytes from the patient. Black arrows indicate the nucleus of the oocyte and hollow arrows indicate the first polar body

**Fig. 4:**
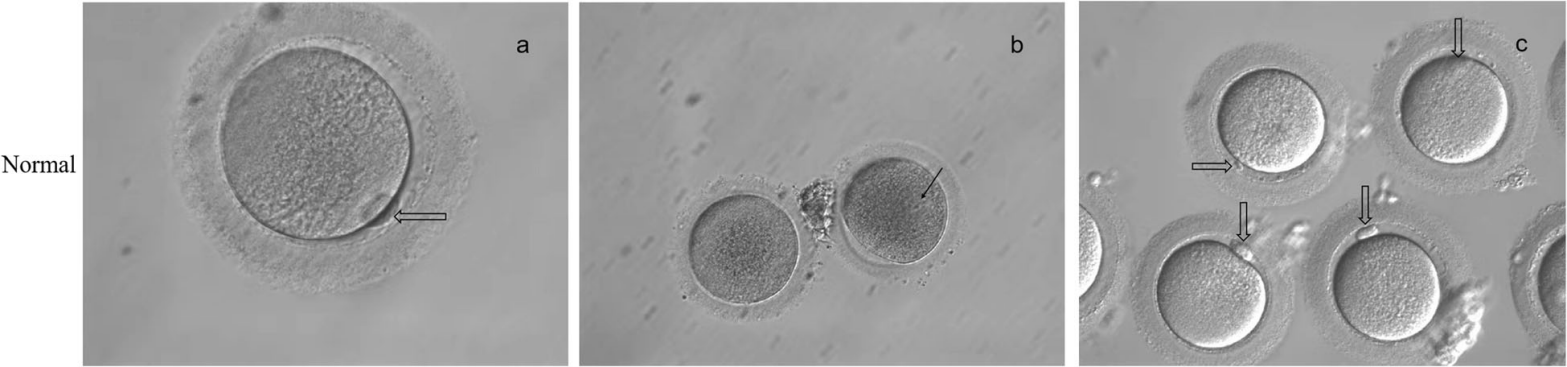
Morphology of oocytes that were retrieved from a healthy individual. **a** One MII oocyte from a healthy individual. **b** One MI oocyte and one GV oocyte from a healthy individual. **c** Four MII oocytes from a healthy individual. Black arrows indicate the nucleus of the oocyte and hollow arrows indicate the first polar body

Transvaginal oocyte retrieval was performed 35-36 h following human chorionic gonadotrophin (HCG) trigger. Oocytes were obtained from the patient. The morphology of oocytes was observed by light microscopy (Figs. 3 and 4).

Although all these immature oocytes were cultured in in vitro mature (IVM) medium for 24 h, they were still immature. So we couldn’t perform fertilization for her. All the cycles were cancelled.

### WES and mutation screening

Genomic DNA was extracted from peripheral venous blood using the QIAamp DNA Blood Mini Kit (Qiagen, Hilden, Germany). All exomes were captured with the SureSelect Human All Exon V5 kit (Agilent, Santa Clara, CA) and then sequenced on the Illumina HiSeq2000 sequencer (Illumina, San Diego, CA). Reads were mapped to the human reference genome (hg19) using BWA − 0.7.10 (Burrows–Wheeler Alignment Tool). Variants including single nucleotide polymorphisms and indels were called by GATK 3.v4 (Genome Analysis Toolkit) and annotated with SnpEff _v4.1. Candidate genes were analyzed on the basis of sequencing results, the family tree, and public databases.

### Validation with sanger sequencing

*TUBB8* was sequenced by Sanger sequencing in three subjects using the primer pair. The sequences of the primer pair were as follows:

Forward primer: 5′-CCCCAACGTGGAAAGGACC-3′;

Reverse primer: 5′-CCCATTCTCAGGAAAGGCAGTAG-3′.

### Online prediction

Public databases, including online forecasting programs, such as 1000 Genomes, ESP6500si, and ExAC Asian population, were used to obtain the frequency of variants. Online prediction programs, including SIFT, Mutation Taster, PolyPhen-2, and PROVEAN, were used to predict the effect of missense mutations on proteins. CLC Sequence Viewer 8 software was used for conservation analysis.

## Results

### Phenotypes of oocytes

Three cycles of ovulation induction therapy (controlled ovarian stimulation) were carried out. During each of the 3 cycles of treatment, oocytes were retrieved from the patient and all oocytes were observed at the GV stage and MI stage under a microscope (Fig. 3). While, after a cycle of ovulation induction treatment, most oocytes of the healthy individual were at the MII stage (Fig. 4). The oocytes retrieved from the patient were all immature at that time, and were not developed to the MII stage after 24–36 h culture in vitro (Table 2).

In the first cycle, 24 immature oocytes were obtained after receiving treatment. After 24 h of culture, all oocytes were still arrested at the GV stage and MI stage.

In the second cycle, 21 oocytes were obtained after receiving treatment. After 24 h of culture in vitro, 14 oocytes were arrested at the GV stage and MI stage, and 7 oocytes degenerated.

In the third cycle, 12 oocytes were obtained after receiving treatment. After 24–36 h of culture, 14 oocytes were arrested at the GV stage and MI stage, and 7 oocytes degenerated.

### WES results

DNA samples from the proband and her parents were used for WES. We selected mutations shared by the three sequenced subjects, and focused on non-synonymous mutations, including nonsense, missense, splice-site, and frameshift mutations. We discarded all variants with a frequency of > 0.1% in public databases (1000 Genomes, ESP6500si, and ExAC Asian population). Because the parents of the proband were cousins and neither of them was affected, we speculated that the inheritance pattern of this mutation was recessive. Therefore, we selected homozygous mutations in the proband. These homozygous mutations included *TUBB8* and *NCOA2* (Table 3). Furthermore, we found some genes related to OOMDs in public databases, including OMIM [11], PubMed [12], GO [13], and MGI databases [14]. In the MGI database and GO database, *NCOA2* plays an important role in chromatin binding activity; RNA polymerase II regulatory region sequence-specific DNA binding activity; and thyroid hormone receptor coactivator activity. *NCOA2* is associated with acute myeloid leukemia. In the PubMed database and OMIM database, *NCOA2* has not been reported to be related to oocyte development. Eventually, the *TUBB8* gene was targeted (Table 3).

**Table 3:** Candidate variants with whole exome sequencing

Gene	Chromosome Position	Variant	1000G(%)	ESP6500si(%)	ExAC_EAS(%)	Online Prediction			
						SIFT	MutationTaster	PolyPhen2	PROVEAN
TUBB8	Chr10:48809	NM_177,987:c.C161T:p.A54V	0	0.01	0.02	D	D	B	N
NCOA2	Chr8:70123918	NM_006540:c.C4259T:p.P1420L	0	0	0	D	D	D	D

*B* predicted to be benign, *D* predicted to be deleterious, *N* predicted to be neutral, *ExAC_EAS* Frequency of corresponding variants in the ExAC Asian population

We identified a *TUBB8* C-T variant (*TUBB8*: NM_177,987: exon 2: c.C161T: p.A54V) at 161th base. This resulted in an amino acid change from alanine to valine at the 54th residue (Table 3).

### Validation with sanger sequencing

*TUBB8* was sequenced by Sanger sequencing in the three subjects to exclude false positive results from WES. We found that the parents of the proband were carriers of the *TUBB8* variant, and the proband was a homozygote of the variant (Fig. 2).

### Online analysis and prediction

This variant was not found in 1000 Genomes, with a frequency of < 0.1% in ESP6500si and ExAC Asian population. Online programs, including MutationTaster and SIFT, predicted this variant to damage protein function. Polyphen-2 and PROVEAN predicted that this variant was benign (Table 3). The *TUBB8* variant was also found to be highly conserved among primates (Fig. 2).

## Discussion

OOMDs can be classified into six subtypes, and different pathogenic genes lead to different genetic and clinical characteristics of different subtypes [4, 5, 7–10, 15]. Mutation of the *TUBB8* gene leads to OOMD2. Inheritance of OOMD2 can be either autosomal dominant or autosomal recessive. Female primary infertility was the clinical trait of OOMD2. The corresponding phenotype includes arrest at MI or MII of oocytes, fertilization failure, stagnation of early embryonic development, and failure of embryo implantation [16]. Expression of TUBB8 protein is unique to oocytes and early embryos. Thus, male carriers of the *TUBB8* mutation are fertile [9].

*TUBB8* is one of the microtubulin family genes. There are nine types of beta-tubulin in mammals. Beta-tubulin can be distinguished mainly by a change in the C-terminal domain affecting specific cell functions [17]. In early embryos, this gene occupies almost all of the expressed beta-tubulin. Microtubules are dynamic polymers composed of alpha/beta-tubulin isodimers [18]. TUBB8 protein has two domains, including a GTPase domain and C-terminal domain.

In previous studies, researchers have found several inheritance patterns of *TUBB8* mutations, including heterozygous mutations [6, 9, 10, 16, 19–21], homozygous mutations [6, 10, 16, 22], compound heterozygous mutations [16, 21], and homozygous deletions [16]. These mutations affect folding and assembly of alpha/beta-tubulin isodimers. This process changes the dynamics of microtubules in vivo, and results in disastrous spindle assembly defects and arrest of oocyte maturation in human oocytes. Some *TUBB8* variants of dominant inheritance have significant negative effects, which interfere with microtubule behavior and meiotic spindle assembly of oocytes, leading to arrest of oocyte maturation and female infertility [19].

We found a consanguineous mating family in which the proband suffered from primary infertility. After a cycle of ovulation induction treatment, most oocytes of the healthy individual were at the MII stage, while oocytes of the patient were at the GV stage or MI stage. After a period of culture in vitro, the oocytes remained immature. We considered that there might be some genetic factors leading to arrest of oocyte development.

We identified a *TUBB8* variant (*TUBB8*: NM_177,987: exon 2: c.C161T: p.A54V) from a family by WES. Because the parents of the proband were cousins and neither of them was affected, we speculated that the inheritance pattern of this mutation was recessive (Fig. 1).

The variant that we found was located in exon 2, and the affected residue(p.A54) was located in the GTPase domain (Fig. 2a). And we referred to a previous study of missense mutations (p.P70L and p.C12Y) located in β-tubulin subunit in the GTPase domain of which their inheritance patterns were also recessive, the two affected residues may influenced folding or protein stability [6]. Thus, the affected residues that we found (p. A54) may influence folding/protein stability. And TUBB8 is an important component of oocyte spindle [9], so the homozygote of the variant that we found might affect spindle assembly, which will result in arrest of oocyte maturation. Heterozygous missense mutations cause arrest of oocyte maturation through dominant-negative effects. In this study, however, the patient with homozygous p.A54V TUBB8 mutations suffered from OOMD2, while her parents with the heterozygous p.A54V missense mutations were fertile. This finding suggested that heterozygous p.A54V mutations could not affect female fertility. Thus, p.A54V has a haploinsufficiency effect than a dominant-negative effect.

We reviewed variants in *TUBB8* that have reported previously (Supplementary Table 1). We found that the inheritance pattern of p.E27_A33del located in exon 2 was recessive [10]. While our newly discovered p.A54V was located in exon 2 and the inheritance pattern was also recessive.

The discovery of this variant started with investigation of the pedigree of OOMD (Fig. 1). Because the proband’s parents were cousins and neither of them was affected, we speculated that the inheritance pattern of this mutation was recessive. Therefore, we selected homozygous mutations from the proband. This helped us to quickly identify this variant of *TUBB8*. Consanguineous mating families are useful for studying mechanisms of genetic diseases without human intervention.

This study elucidated the cause of oocyte maturation defects. The results confirmed that mutations of *TUBB8* contributed genetically to OOMD2 and expanded the mutation spectrum of *TUBB8*.

## Conclusions

A rare variant of *TUBB8* (*TUBB8*: NM_ 177,987: exon 2:c.C161T: p.A54V) was found in a Chinese consanguineous mating family with OOMD2 by WES and Sanger sequencing. The homozygote of this variant might affect spindle assembly, which will cause arrest of oocyte maturation and lead to primary infertility. The variant (*TUBB8*: NM_ 177,987: exon 2:c.C161T: p.A54V) in the public database is rare, and this site is highly conserved among primates. Our findings confirmed that mutations of *TUBB8* contributed genetically to OOMD2. Our findings expanded the mutation spectrum of *TUBB8* causing OOMD2 and provided a basis for targeted therapy in the future.

## Supplementary information

**Additional file 1 **: **Supplementary Table 1.** Variants of *TUBB8* reported in previous studies.

## Data Availability

Not applicable.
