# Supplementary material for: Rare homozygous mutation in TUBB8 associated with oocyte maturation defect-2 in a consanguineous mating family
Source: J Ovarian Res. 2020 Apr 21;13:42. doi: 10.1186/s13048-020-00637-4 (PMC7175565; doi:10.1186/s13048-020-00637-4)
Supplement: Supplementary file 1 — Additional file 1 : Supplementary Table 1. Variants of TUBB8 reported in previous studies. [file 13048_2020_637_MOESM1_ESM.docx]

Supplementary Table 1 Variants of *TUBB8* reported in previous studies.

| variant | Amino acid alteration | exon | DOMAIN | Inheritance pattern | PPH2 | PROVEAN | Hetero-/Homo-/Compound heterozygote | Variant type | reference |
| --- | --- | --- | --- | --- | --- | --- | --- | --- | --- |
| c.10A>C | p.(I4L) | 1 | 1 | Unknown | Benign | N | Hetero- | Missense | [1] |
| c.181C>A | p.(P61T) | 3 | 1 | Unknown | PB | D | Hetero- | Missense |  |
| c.322G>A | p.(E108K) | 4 | 1 | AD | PB | D | Hetero- | Missense |  |
| c.426dupG | p.(T143Dfs*12) | 4 | 1 | AR | / | / | Hetero- | Frameshift insertion | |
| c.292G>A | p.(G98R) | 4 | 1 | Unknown | PB | D | Hetero- | Missense |  |
| c.527C>T | p.(S176L) | 4 | 1 | Unknown | PB | D | Hetero- | Missense |  |
| c.523G>A | p.(V175M) | 4 | 1 | Unknown | PB | N | Hetero- | Missense |  |
| c.600T>G | p.(F200L) | 4 | 1 | Unknown | Benign | D | Hetero- | Missense |  |
| c.763G>A | p.(V255M) | 4 | 1.5 | AD | PB | N | Hetero- | Missense |  |
| c.721C>T | p.(R241C) | 4 | 1.5 | AR | PB | D | Homo- | Missense |  |
| c.735G>C | p.(Q245H) | 4 | 1.5 | Unknown | PB | D | Hetero- | Missense |  |
| c.722G>A | p.(R241H) | 4 | 1.5 | Unknown | PB | D | Hetero- | Missense |  |
| c.1073C>T | p.(P358L) | 4 | 2 | AD | PB | D | Hetero- | Missense |  |
| c.1000C>G | p.(Q334E) | 4 | 2 | AD | PB | N | Hetero- | Missense |  |
| c.883G>C | p.(D295H) | 4 | 2 | Incomplete dominance | PB | D | Hetero- | Missense |  |
| c.1099T>C | p.(F367L) | 4 | 2 | AD | PB | D | Hetero- | Missense |  |
| c.1072C>G | p.(P358A) | 4 | 2 | Unknown | PB | D | Hetero- | Missense |  |
| c.1061G>A | p.(C354Y) | 4 | 2 | Unknown | PB | D | Hetero- | Missense |  |
| c.1057G>A | p.(V353I) | 4 | 2 | Unknown | Benign | N | Hetero- | Missense |  |
| c.1270C>T | p.(Q424*) | 4 | 2.5 | AR | / | / | Homo- | Non-sense |  |
| c.1286C>T | p.(T429M) | 4 | 2.5 | Incomplete dominance | PB | N | Compound heterozygote | Missense |  |
| c.1301_1327del | p.(434_442del) | 4 | 2.5 | Incomplete dominance | / | D | Compound heterozygote | In-frame deletion | |
| c.1205dupG | p.(M403Hfs*3) | 4 | 2.5 | AR | / | / | Homo- | Frameshift insertion | |
| c.1249G>T | p.(D417Y) | 4 | 2.5 | Unknown | PB | D | Hetero- | Missense |  |
| c.1171C>T | p.(R391C) | 4 | 2.5 | Unknown | PB | D | Hetero- | Missense |  |
| c.1228G>A | p.(E410K) | 4 | 2.5 | Unknown | PB | N | Hetero- | Missense |  |
| c. 80_100del | p.E27_A33del | 2 | 1 | AR |  |  | Homo- | In-frame deletion | [2] |
| c. 527C>T | p.S176L | 4 | 1 | Unknown |  |  | Hetero- | Missense |  |
| c. 628A>G | p.I210V | 4 | 1 | Unknown |  |  | Hetero- | Missense |  |
| c. 853A>C | p.T285P | 4 | 1 | Unknown |  |  | Hetero- | Missense |  |
| c. 1043A>G | p.N348S | 4 | 1 | Unknown |  |  | Hetero- | Missense |  |
| c. 426_427insG | p.T143Dfs*12 | 4 | 1 | AR |  |  | Homo- | Frameshift insertion | |
| c. 713C>T | p.T238M | 4 | 1.5 | AD |  |  | Hetero- | Missense |  |
| c. 763G>A | p.V255M | 4 | 1.5 | Unknown |  |  | Hetero- | Missense |  |
| c. 784C>T | p.R262Q | 4 | 2 | Unknown |  |  | Hetero- | Missense |  |
| c. 5G>A | p.R2K | 1 | "-1" | AD |  |  | Hetero- | Missense | [3] |
| c. 527C>T | p.S176L | 4 | 1 | Unknown |  |  | Hetero- | De novo |  |
| c. 686T>C | p.V229A | 4 | 1 | AD |  |  | Hetero- | Missense |  |
| c. 785G>A | p.R262Q | 4 | 2 | Unknown |  |  | Hetero- | Missense |  |
| c. 900G>A | p.M300I | 4 | 2 | AD |  |  | Hetero- | Missense |  |
| c. 1088T>C | p.M363T | 4 | 2 | AD |  |  | Hetero- | Missense |  |
| c. 1249G>A | p.D417N | 4 | 2 | AD |  |  | Hetero- | Missense |  |
| c. 5G>T | p.R2M | 1 | 1 | AD | PB | N | Hetero- | Missense | [4] |
| c. 10A>C | p.I4L | 1 | 1 | AD | benign | N | Hetero- | Missense |  |
| c. 35G>A | p.C12Y | 1 | 1 | AR | benign | D | Homo- | Missense |  |
| c. 209C>T | p.P70L | 3 | 1 | AR | PB | D | Homo- | Missense |  |
| c. 580G>A | p.E194K | 4 | 1 | Unknown | PB | D | Hetero- | Missense |  |
| c. 613G>A | p.E205K | 4 | 1 | Unknown | PD | D | Hetero- | Missense |  |
| c. 990G>A | p.M330I | 4 | 2 | AD | benign | D | Hetero- | Missense |  |
| c. 1057G>A | p.V353I | 4 | 2 | Unknown | benign | N | Hetero- | Missense |  |
| c. 1245G>A | p.M415I | 4 | 2 | Unknown | benign | N | Hetero- | Missense |  |
| Exon 1–4 deletion | NA | 1–4 |  | AR | NA | NA | Homo- | Whole deletion | |
| c. 5G>T | p.R2M | 1 | 1 | AD | D | D(SIFT) | Hetero- | Missense | [5] |
| c. 535G>A | p.V179M | 4 | 1 | AD | D | D(SIFT) | Hetero- | Missense |  |
| c. 292G>A | p.G98R | 4 | 1 | Unknown | D | D(SIFT) | Hetero- | Missense | [6] |
| c.322G > A | p.Glu108Lys | 4 | 1 | Unknown | PD | D(SIFT) | Hetero- | Missense | [7] |
| c.1054G>T | p.A352S | 4 | 2 | AD | PD | disease-causing(Mutation Taster) | Hetero- | Missense | [8] |
| c.161C>T | p.A54V | 2 | 1 | AR | benign | N | Homo- | Missense | this study |

PB, Probably damaging; D, Deleterious; N, Neutral;

Domain "-1"：Location in front of GTPase domain;

Domain 1：Tubulin FtsZ family, GTPase domain;

Domain 1.5: Location between GTPase domain and Tubulin C-terminal domain;

Domain 2: Tubulin C-terminal domain;

Domain 2.5: Location behind Tubulin C-terminal domain.

References

1. Chen B, Wang W, Peng X*, et al.* The comprehensive mutational and phenotypic spectrum of TUBB8 in female infertility. Eur J Hum Genet 2019; **27**: 300-307. DOI: 10.1038/s41431-018-0283-3

2. Feng R, Yan Z, Li B*, et al.* Mutations in TUBB8 cause a multiplicity of phenotypes in human oocytes and early embryos. J Med Genet 2016; **53**: 662-671. DOI: 10.1136/jmedgenet-2016-103891

3. Feng R, Sang Q, Kuang Y*, et al.* Mutations in TUBB8 and Human Oocyte Meiotic Arrest. N Engl J Med 2016; **374**: 223-232. DOI: 10.1056/NEJMoa1510791

4. Chen B, Li B, Li D*, et al.* Novel mutations and structural deletions in TUBB8: expanding mutational and phenotypic spectrum of patients with arrest in oocyte maturation, fertilization or early embryonic development. Hum Reprod 2017; **32**: 457-464. DOI: 10.1093/humrep/dew322

5. Huang L, Tong X, Luo L*, et al.* Mutation analysis of the TUBB8 gene in nine infertile women with oocyte maturation arrest. Reprod Biomed Online 2017; **35**: 305-310. DOI: 10.1016/j.rbmo.2017.05.017

6. Wang AC, Zhang YS, Wang BS*, et al.* Mutation analysis of the TUBB8 gene in primary infertile women with arrest in oocyte maturation. Gynecol Endocrinol 2018; **34**: 900-904. DOI: 10.1080/09513590.2018.1464138

7. Yuan P, Zheng L, Liang H*, et al.* A novel mutation in the TUBB8 gene is associated with complete cleavage failure in fertilized eggs. J Assist Reprod Genet 2018; **35**: 1349-1356. DOI: 10.1007/s10815-018-1188-3

8. Xiang J, Wang W, Qian C*, et al.* Human oocyte maturation arrest caused by a novel missense mutation in TUBB8. J Int Med Res 2018; **46**: 3759-3764. DOI: 10.1177/0300060518778638
